# Visualization of Active Glucocerebrosidase in Rodent Brain with High Spatial Resolution following *In Situ* Labeling with Fluorescent Activity Based Probes

**DOI:** 10.1371/journal.pone.0138107

**Published:** 2015-09-29

**Authors:** Daniela Herrera Moro Chao, Wouter W. Kallemeijn, Andre R. A. Marques, Marie Orre, Roelof Ottenhoff, Cindy van Roomen, Ewout Foppen, Maria C. Renner, Martina Moeton, Marco van Eijk, Rolf G. Boot, Willem Kamphuis, Elly M. Hol, Jan Aten, Hermen S. Overkleeft, Andries Kalsbeek, Johannes M. F. G. Aerts

**Affiliations:** 1 Department of Medical Biochemistry, Academic Medical Center, Amsterdam, The Netherlands; 2 Department of Endocrinology and Metabolism, Academic Medical Center, Amsterdam, The Netherlands; 3 Netherlands Institute for Neuroscience, Amsterdam, The Netherlands; 4 Department of Translational Neuroscience, Brain Center Rudolf Magnus, University Medical Center Utrecht, Utrecht, The Netherlands; 5 Swammerdam Institute for Life Sciences, Center for Neuroscience, University of Amsterdam, Amsterdam, The Netherlands; 6 Department of Pathology, Academic Medical Center, Amsterdam, The Netherlands; 7 Department of Bio-organic Synthesis, Leiden institute of Chemistry, Leiden, The Netherlands; 8 Department of Biochemistry, Leiden Insitute of Chemistry, Leiden, The Netherlands; UCL Institute of Neurology, UNITED KINGDOM

## Abstract

Gaucher disease is characterized by lysosomal accumulation of glucosylceramide due to deficient activity of lysosomal glucocerebrosidase (GBA). In cells, glucosylceramide is also degraded outside lysosomes by the enzyme glucosylceramidase 2 (GBA2) of which inherited deficiency is associated with ataxias. The interest in GBA and glucosylceramide metabolism in the brain has grown following the notion that mutations in the GBA gene impose a risk factor for motor disorders such as α-synucleinopathies. We earlier developed a β-glucopyranosyl-configured cyclophellitol-epoxide type activity based probe (ABP) allowing *in vivo* and *in vitro* visualization of active molecules of GBA with high spatial resolution. Labeling occurs through covalent linkage of the ABP to the catalytic nucleophile residue in the enzyme pocket. Here, we describe a method to visualize active GBA molecules in rat brain slices using *in vivo* labeling. Brain areas related to motor control, like the basal ganglia and motor related structures in the brainstem, show a high content of active GBA. We also developed a β-glucopyranosyl cyclophellitol-aziridine ABP allowing *in situ* labeling of GBA2. Labeled GBA2 in brain areas can be identified and quantified upon gel electrophoresis. The distribution of active GBA2 markedly differs from that of GBA, being highest in the cerebellar cortex. The histological findings with ABP labeling were confirmed by biochemical analysis of isolated brain areas. In conclusion, ABPs offer sensitive tools to visualize active GBA and to study the distribution of GBA2 in the brain and thus may find application to establish the role of these enzymes in neurodegenerative disease conditions such as α-synucleinopathies and cerebellar ataxia.

## Introduction

Gaucher disease (GD) is caused by a recessively inherited deficiency of the lysosomal hydrolase glucocerebrosidase (GBA) encoded by the GBA gene. The enzyme deficiency results in lysosomal accumulation of its glycosphingolipid substrate, glucosylceramide (GlcCer) [1]. In contrast to other, more complex glycosphingolipids and galactosylceramide, GlcCer is present both in the cytosolic and luminal leaflets of membranes. Degradation of GlcCer in cells is therefore not restricted to the lysosomes, but also partly takes place through the action of the non-lysosomal β-glucosylceramidase 2 (GBA2) [2]. GD patients generally show a massive GlcCer accumulation in lysosomes of tissue macrophages in the spleen, liver and bone marrow, leading to characteristic hepatosplenomegaly, thrombocytopenia, anemia and leukopenia. Prominent pathology of the central nervous system does not develop in most GD patients. This non-neuropathic variant is commonly referred to as type 1 GD. More severely affected GD patients, so-called type 2 and 3 GD, do present neurological symptoms stemming from neuronal degeneration [1]. Postmortem evaluations of brain from GD mice and type 2 and 3 GD patients have revealed α-synuclein (Lewy body) deposits in brainstem and midbrain structures like the pyramidal tracts and the pontine nucleus (Po), the basal ganglia (striatum, substantia nigra (SN), globus pallidus (GP)), the subthalamic nucleus, the cerebellum and the hypothalamus [3,4]. Neuronal loss in type 2 and 3 GD patients is most evident in the cortex and in dopaminergic neurons of the SN [4]. Of note, GD patients, and even carriers of mutations in the GBA gene, are at increased risk for developing motor disorders such as Parkinsonism [5–10]. Likewise, in mice reduced GBA activity leads to accumulation of α-synuclein species [11, 12]. In mouse models of GD and Parkinson disease, introduction of active GBA in the brain by lentiviral gene therapy has a beneficial effect [13, 14]. The lysosomal integral membrane protein type-2 (LIMP-2) mediates the transport of newly formed GBA to lysosomes [15]. Brain of LIMP-2–deficient mice with reduced GBA activity shows increased α-synuclein deposits causing neurotoxicity of dopaminergic neurons as well as apoptotic cell death and inflammation [16]. At present, therapeutic introduction of GBA, or LIMP-2, in the brain is still not feasible. Current enzyme replacement therapy (ERT) of GD patients is based on two-weekly intravenous infusions with macrophage-targeted recombinant glucocerebrosidase [17]. Although ERT results in improvements in the viscera, neurological manifestations in type 2 and 3 GD patients are not prevented by the intravenous enzyme infusions. This lack of effect is ascribed to the poor passage of ERT enzyme across the blood brain barrier. An alternative approach for modulating GlcCer metabolism in the brain may be offered by the use of brain-permeable small compounds inhibiting GlcCer synthase [18]. The presently registered inhibitors for substrate reduction therapy of GD do not prevent neurological manifestations due to poor brain permeability [19]. Development of better brain-permeable inhibitors of GlcCer synthase would be therefore required for this and is actively pursued [20].

Given the importance of abnormalities in GBA for neurological manifestations in type 2 and 3 GD patients, as well as its possible role in onset of α-synucleinopathies, far more detailed information of localization of active GBA in the brain is desired. The same holds for the enzyme GBA2. In recent years, we developed a new toolbox to study active GBA and GBA2 molecules within living cells and laboratory animals. Detection is based on β-glucopyranosyl-configured cyclophellitol-type ABPs that specifically and covalently link to the catalytic nucleophile in the enzymatic pocket of retaining β-glucosidases, employing the double-displacement mechanism for catalysis. The developed ABPs are equipped with a green- or red fluorescent BODIPY group and allow ultrasensitive visualization of the active enzyme molecules *in vitro* and *in vivo* [21–26]. The first class of ABPs consists of compounds with a cyclophellitol β-epoxide moiety linked to a fluorescent BODIPY group [20, 24]. Covalent binding to the catalytic glutamate 340 of GBA proceeds by the same mechanism as that of conduritol B-epoxide (CBE) [20, 24]. The cyclophellitol β-epoxide type ABPs with distinct fluorescent BODIPY moieties specifically label active GBA molecules, and are for convenience here referred to as inhibodies. The second class of ABPs consists of β-aziridine cyclophellitol with a linked fluorescent BODIDY group. Contrary to the cyclophellitol β-epoxide ABPs, the β-aziridine cyclophellitol-type structures label the entire class of retaining β-glucosidases, including GBA, GBA2, GBA3, and lactase/phoridzin hydrolase (LPH) [23, 27]. This broad-specific ABP is here referred to as Anybody. A drawback of all BODIPY-containing ABPs is their inability to penetrate the brain [21]. Following intravenous or intraperitoneal administration of ABPs the target enzymes (GBA and/or GBA2) are efficiently labeled in all tissues with exception of the eye and brain [21–23, 28].

The primary objective of this study was to establish whether intracerebroventricular (i.c.v.) infusion of cyclophellitol β-epoxide ABPs offers a suitable method to specifically visualize *in vivo* active GBA molecules with high spatial resolution. The secondary objective was to study the feasibility of labeling active GBA2 in the intact brain by i.c.v. infusion of the β-aziridine cyclophellitol ABP. We here describe the outcome of the investigation and discuss the observed distribution of active GBA and GBA2 molecules in rat brain.

## Materials & Methods

### Materials

The various ABPs (cyclophellitol β-epoxide MDW933 (Inhibody Green); cyclophellitol β-epoxide MDW941 (Inhibody Red); β-aziridine cyclophellitol MDW1044 (Anybody Green); β-aziridine cyclophellitol JJB75 (Anybody Red)) were synthesized exactly as described earlier [21, 23]; conduritol B-epoxide (CBE) was purchased from Sigma (St Louis, USA).

### Animals

Wistar rats (20 male individuals, 8–10 weeks old) were used for i.c.v. administration of ABPs. For each ABP administration group, 4 animals were used. Each animal was kept in an individual cage at constant temperature (23°C ± 2°C), submitted to a 12/12h light/dark cycle. They were exposed to *ad libitum* food and water before and after the experimental procedures. All animal protocols were approved by the Institutional Animal Welfare Committee of the Academic Medical Center at the University of Amsterdam, Amsterdam (The Netherlands).

### Methods

#### *In vivo* i.c.v. administration of fluorescent ABPs to the brain

Wistar rats were infused i.c.v. with fluorescent ABPs. Animals were anesthetized with a mixture of 0.4 ml/kg Dormicum (Roche, Almere, The Netherlands) and 0.8 ml/kg Hypnorm (Janssen, High Wycombe, Buckinghamshire, UK). Intracerebroventricular stainless steel guide cannulas were implanted in the lateral ventricle using the following stereotaxic coordinates: AP -0.9, L +2.0 and V -3.4, tooth bar (-.2.5mm). Analgesic Buprecare (Recipharm, Lancashire, UK) was administered after the animals woke up from the surgery. After a recovery period of 7 days (body weight recovery to the pre-surgery point), a needle connected to a tube was introduced in the guide cannula and PBS solutions containing 1 mM of different ABPs (MDW933 and MDW941) were infused with a rate of 1 μl/min for 10 minutes. After 4 hours, the animals were sacrificed by CO_2_ euthanasia and transcardially perfused with 250 ml of 0.9% (w/v) saline solution. In indicated experiments, animals were first i.c.v. infused with CBE (1 mM in PBS) at 1 μl/min for 10 minutes. After 4 hours of recovery, the animals received i.c.v. Anybody Red (JJB75) at the same concentration and rate as above. After another 4 hours, the animals were sacrificed by CO_2_ euthanasia and transcardially perfused with 250 ml 0.9% (w/v) saline solution. Brains were isolated and immediately frozen for further biochemical and histological analysis.

#### Analysis of brain sections

Rat brains were cut in 30 μm slices with a cryostat and attached to SuperFrost slides (Thermo Scientific, Waltham, USA). All following steps were in the dark to protect the fluorescence of the ABPs. Slides were extensively washed in 0.01 M Tris-buffered saline (TBS) to remove non-specific fluorescence and were covered in DAPI (Vector Lab, Burlingame, USA) mounting media.

For immunohistological analysis, brain slices were extensively washed in TBS, next incubated overnight in TBS containing 0.5% (v/v) Triton X-100, 0.025% (v/v) gelatin and supplemented with one of the following primary antibodies (1:1000): rabbit anti-rat LAMP1 (Millipore, MA, USA), Cathepsin D (Millipore, MA, USA), NeuN (Millipore, MA, USA), GFAP (DAKO, Heverlee, Belgium), Cd11b (Sigma, St Louis, USA), Iba1 (Wako, California, USA), GBA (Sigma, St Louis, USA), Calbindin D 28 K (Millipore, MA, USA) and GBA2 (see description below). Hereafter, slides were incubated with appropriate secondary antibodies (1:1000) conjugated with fluorescent dyes; either donkey anti-rabbit Alexa488 or Alexa647 (Life Technologies, Paisley, UK) for 2 hours. After rinsing slides three times in TBS, these were mounted and covered with DAPI to be observed in a confocal laser-scanning microscope (Leica SP5). The rabbit polyclonal anti-mouse GBA2 antibodies were generated using the Double XP program from Eurogentec (Liege, Belgium), with the GBA2 peptides CGSPEDSGPQDEPSY and GRYYNYDSSSHPQSR. Final bleeds were affinity purified using the same peptides.

For the isolation of rat brain areas, frozen brains were dissected in 10 areas. The cerebellum, brainstem and spinal cord were isolated from the rest of the brain by separating the hindbrain from the cortical lobes at the level of inferior colliculus. The pons was separated and the brainstem cut slightly lower of the obex to separate it from the spinal cord. For the hippocampus, small curved blunt forceps were introduced between the cerebral hemispheres in a closed position, slowly opening the forceps; the cortex was separated from the hippocampus. The hippocampus was next dissected. For the dissection of the cerebellum, slices of 300 μm were made from the whole cerebellum and with a steel needle the area of the cerebellar cortex was taken per slice. The rest of the cerebellum from each slice was considered cerebellum. For the cingular cortex, putamen and globus pallidus, slices of 300 μm from the enlargement of the lateral ventricle and beginning of the putamen were made. Using a steel needle, the cingular cortex 1 and 2 and the anterior putamen alone were dissected, and subsequently the putamen and globus pallidus. Before hypothalamus dissection, the frontal part of the brain was cut coronally for dissection of the forebrain areas. After that, blunt curved forceps were introduced at the level of the mammillary nucleus and the hypothalamus was separated from the rest of the brain. Finally, the thalamus was dissected above the border of the missing hypothalamus and the anterior commissure using a steel needle.

#### Analysis of ABP labeling by gel electrophoresis and fluorescence scanning

Lysates of rat dissected brain areas were made by homogenizing the frozen material in 25 mM potassium phosphate buffer, pH 6.5, supplemented with 0.1% (v/v) Triton X-100 and protease inhibitor cocktail (Roche, Indianapolis, USA). The electrophoresis in sodium dodecyl sulfate containing 7.5% polyacrylamide gels and quantitative scanning of the gels was exactly performed as earlier described [21, 23]. Protein was determined using the Pierce BCA Protein Assay kit (Thermo Scientific, Paisley, UK).

#### Enzymatic activity assays

For determination of GBA-associated β-glucosidase activity, 3.75 mM 4-methylumbelliferyl-β-D-glucopyranoside (Sigma, St Louis, USA) was used as artificial substrate at 37°C, in 150 mM citric acid-Na_2_HPO_4_ at pH 5.2, in the presence of 0.2% (w/v) sodium taurocholate, 0.1% (v/v) Triton X-100 and 0.1% (w/v) bovine serum albumin (BSA) [29]. Determination of GBA2-associated β-glucosidase activity occurred in 150 mM citric acid-Na_2_HPO_4_ at pH 5.8 in the presence of the same substrate and 0.1% (w/v) BSA [2]. β-Hexosaminidase activity was measured with 1.97 mM 4-MU-*N*-acety-β-D-glucosaminide in 150 mM citric acid-Na_2_HPO_4_, pH 4.0, supplemented with 0.1% (w/v) BSA. All enzymatic reactions were stopped with excess NaOH-glycine (pH 10.6) and fluorescence of liberated 4-methylumbelliferone was measured with a fluorimeter LS55 (Perkin Elmer) using λex 366 nm and λem 445 nm.

#### Isolation of microglia and astrocytes

Wistar rats were anesthetized and perfused with Hank’s Balanced Salt Solution (HBSS) from Life Technologies (Carlsbad, USA). Their brains were dissected in forebrain and hindbrain (brainstem, spinal cord and midbrain including substantia nigra) and kept in ice-cold HBSS. After the isolation of the brain, the hindbrain was cut at the level of the inferior colliculus and an extra slice was made with a steel blade to also obtain the substantia nigra. After mechanical homogenization using a steel blade, the tissue was digested with papain (Worthington, Lakewood, USA) at a final concentration of 8 U/ml with DNAse I at 80 units/μl (Sigma, St Louis, USA). The mixture was centrifuged at 200× *g* for 15 minutes and the pellet was triturated to obtain a single cell suspension. Myelin was removed by a 90% Percoll demyelination and a FACS sorting procedure, using myelin removal beads (MiltenylBiotec, Bergisch Gladbach, Germany). For a detailed description of the isolation procedure see Orre et al [30].

For the sorting by FACS cells were incubated with a custom-made anti-rabbit polyclonal GLT1 antibody (Biomatik, Wilmington, USA) (1:100), a rabbit anti-rat Cd11b-APC antibody (eBioscience, San Diego, USA) (1:200) and an Fc-receptor blocker CD32 (BD Pharmigen, San Diego, USA) for 30 minutes. After washing of cells, these were incubated with a biotinylated antibody (Jackson Immunoresearch, Suffolk, UK). After additional washings, cells were again incubated with streptavidin APC-Cy7 (Biolegend, San Diego, USA) and next resuspended in GKN/BSA buffer (8 g/L NaCl, 0.4 g/L KCl, 1.77 g/L Na_2_HPO_4_•2H_2_O, 0.69 g/L NaH_2_PO_4_•H_2_O, 2 g/l d-(1)-glucose, pH 7.4). To differentiate viable from dead cells, 7AAD (BD Bioscience, San Diego, USA) was added. Cells were sorted using a BD FACS After gating the dead cells based on 7AAD expression, astrocytes were sorted based on GLT1+/CD11b- expression, microglia were sorted based on CD11b+ expression and the rest of the cells were sorted as non-GLT1+/CD11b+ cells. The sorted cells were lysed immediately after and either used for RNA extraction or cells were frozen as a dry pellet for subsequent labelling with ABP.

#### RNA extraction and RT PCR

The sorted cells were lysed and total RNA was extracted using a Nucleospin II extraction kit (Macherey-Nagel GmbH, Duren, Germany). cDNA was synthesized according to the Invitrogen cDNA synthesis kit. Gene expression analysis was performed using a Bio Rad MyIQ Real Time PCR detection system. The expression levels were normalized to P0 and GADPH expression levels. For the confirmation of the cell types, we used the primers presented in Table 1.

**Table 1:** Primers used for RT PCR of mRNA of specific genes expressed in astrocytes and microglia.

**Astrocytes markers**	
***Glial high affinity glutamate transporter*, *member 2 (Slc1a2)***	FWD: 5'-agtgtgtctatgccgcacac-3'
	REV: 5'-ggctgagaatcgggtcatta-3'
***Aldehyde dehydrogenase 1 family*, *member L1 (Aldh1l1)***	FWD: 5'-gcaccttttggaggattcaa-3'
	REV: 5'-ggcaacagggtacaggagaa-3'
***Fibroblast growth factor receptor 3 (Fgfr3)***	FWD: 5'-cgttgtagagagggctggac-3'
	REV: 5'-gaggcttgccacataagagc-3'
**Microglia markers**	
***Allograft inflammatory factor 1 (Aif1)***	FWD: 5'-aatgatgctgggcaagagat-3'
	REV: 5'-cctccaattagggcaactca-3'
***Integrin*, *alpha M (Itgam)***	FWD: 5'-cgtttgtgaaggctcagaca-3'
	REV: 5'-gtcgtttgaaaaagccaagc-3'
***Integrin*, *beta 2 (Itgb2)***	FWD: 5'-agtcccagtggaacaacgac-3'
	REV: 5'-ggaggggtctcctctgactc-3'
***Chemokine (C-X3-C motif) receptor 1 (Cx3cr1)***	FWD: 5'-gcctcaacccctttatctacg-3'
	REV: 5'-tgctcctctgggactctgtt-3'
**Oligodendrocytes markers**	
***Myelin basic protein (Mbp)***	FWD: 5'-ccgaggagagtgtgggttta-3'
	REV: 5'-ctggagggtttgtttctgga-3'
***Myelin-associated oligodendrocyte basic protein (Mobp)***	FWD: 5'-caggaccacacgatttctt-3'
	REV: 5'-ggaggtcagagcacaagagg-3'
**Neuronal markers**	
***Syntaxin 1A (Stx1a)***	FWD: 5'-gctctgaaaagccaggtgtc-3'
	REV: 5'-cagctggctgctgaatactg-3'
***Neurofilament*, *medium polypeptide (Nefm)***	FWD: 5'-agtggtggtgaccaagaagg-3'
	REV: 5'-caccagcttctcctcaaagg-3'

#### ABP labeling of enzymes in isolated astrocytes and microglia

To label GBA in the astrocytes and microglia, cell pellets were incubated with 200 nM MDW933 (Inhibody green) in McIlvaine buffer (150 mM citric acid-Na_2_HPO_4_, pH 5.2, 0.2% (w/v) sodium taurocholate, 0.1% (v/v) Triton X-100) with protease inhibitors (Roche Diagnostics Ltd, Indianapolis, USA) for 30 min at 37°C. Samples were denatured with Laemmli loading buffer (1 M Tris-HCl, pH 6.8, 0.1% (w/v) bromophenol blue, 60% (w/v) glycerol, 100 mM DTT) and boiled for 4 min at 100°C. SDS-PAGE (electrophoresis in sodium dodecylsulfate (SDS) 7.5% polyacrylamide gels) was performed and followed by fluorescence scanning as earlier described [21].

#### Western Blot Analysis

Rat brain area homogenates (100μg) were subjected to electrophoresis on 7.5% SDS-polyacrylamide gels and then transferred to nitrocellulose membranes (Whatman, Dassel, Germany) using an electroblotting apparatus (Bio-Rad Laboratories, Hercules, CA). The blots were blocked in 2% (w/v) non-fat dried milk in TBST buffer (10 mM Tris-HCl, pH 8.0, 150 mM NaCl, 0.05% (v/v) Tween-20) and incubated with α-GBA (Sigma, St. Louis, USA), α-GBA2 (Eurogentec custom made) and α-Tubulin (Cedarlane Laboratories, Ontario, Canada) primary antibodies diluted in blocking buffer, overnight at 4°C. Blots were washed for 30 minutes in TBST. After washing, the membranes were incubated with matching IRDye-conjugated secondary antibodies (Westburg BV, Leusden, Netherlands) for 1 hour at room temperature. Blots were scanned on an Odyssey image scanner (GE Healthcare).

#### Confocal image analysis

Images were made with a Confocal SP5 Leica, using an excitation wavelength of 488 nM for Alexa488 and MDW933, 561 nM for Alexa594, MDW941 and JJB75, and 652 nM for Alexa647. A 63× objective was used for image capture, and a further 3× zoom was used for single-cell images.

#### Live imaging of brain tissue *ex vivo*

Seven male C57BL/6J mice were anesthetized in an isofluorane chamber and once they were unconscious, decapitation was performed. Their brains were isolated and briefly perfused with ice-cold sucrose cutting solution (72 mM sucrose, 22 mM glucose, 2.6 mM NaHCO_3_, 83 mM NaCl, 2.5 mM KCl, 3.3 mM MgSO_4_ mM and 2 mM CaCl_2_). Directly after, the brain was placed in a vibratome (Thermo scientific, Microm HM 650V) in the aforementioned sucrose solution and gassed with 5% CO_2_ / 95% O_2_. Coronal brain slices of 400 μm thick were made of the midbrain, from in front and behind the substantia nigra, and next transferred to a water bath at 37°C in artificial CSF (118 mM NaCl, 2.5 mM KCl, 26 mM NaHCO_3_, 1 mM NaH_2_PO_4_, 10 mM glucose, 4 mM MgCl_2_, 4 mM CaCl_2_, pH 7.4, gassed with 5% CO_2_ / 95% O_2_) for 30 minutes. Next, the slices were transferred to a petri dish in a chamber for live-cell imaging with 5% CO_2_ / 95% O_2_ and a constant temperature of 37°C. Slices were fixed using a tissue adhesive (3M, Vetbond, Minnesota, USA) in one corner of the slice away from the substantia nigra area. Subsequently, a drop of DAPI mounting media was added to be able to visualize the cells using a 40× magnitude objective in a Zeiss Axiovert 200 M fluorescence microscope. Twenty-second interval time-lapse images were made as a basal measurement for 10 minutes. Next, the slices were incubated with 5 nM MDW941 (Inhibody red) and immediately time-lapse imaging was again resumed. Next, the media were removed and slices were directly frozen for further histological analysis. In some experiments, additional slices were first incubated with CBE (650 μM) for 60 minutes prior to time-lapse imaging as described above.

After the time-lapse imaging, mouse brain slices (30 μm) were stained with primary rabbit anti-mouse antibody against tyrosine hydroxylase (TH) (Abcam, Cambridge, UK). Washings and secondary antibody staining was performed as described above.

#### Histochemical detection of GBA2 in Purkinje cells of mouse brain

Mice were anaesthetized using FFM mix (hypnorm/dormicum/distilled water, 1:1:2 volume). Brains were dissected. Formalin-fixed, paraffin-embedded tissue was sectioned at 4-μm thickness, dried overnight at 37°C, deparaffinized in xylene and rehydrated in graded alcohol. Endogenous peroxidase activity was blocked by incubation for 10 min in methanol containing 0.3% H2O2. Sections were rehydrated and slides were heated in 0.04 M citrate, 0.12 M phosphate, pH 5.8, for 10 min at 121°C. After washing, the sections were incubated with primary antibody being either polyclonal rabbit IgG anti-mouse GBA2 or rabbit IgG anti-calbindin D-28K (PC253L, 1:1,000; Calbiochem, San Diego, CA, USA), in Antibody Diluent (ImmunoLogic, Klinipath, Duiven, The Netherlands) for 16 hours at 4°C, washed, and incubated with secondary antibody. Sections were counterstained with methyl green and mounted with VectaMount (Vector Laboratories). Analysis was performed using brightfield microscopy (Leica DM5000B) with an HC PLAN APO 20x/0.70 objective.

#### Statistical analysis

The FACS sorted population experiments were analyzed by two-way ANOVA, followed by the Bonferroni post-hoc test. For the gene-expression of isolated cells, one-way ANOVA was performed, followed by the Tukey post-hoc test. For both tests, a probability of p<0.05 was set as significant. The data was analyzed using a SPSS version 15 statistical package.

## Results

### Visualization of active GBA by i.c.v. administration of with cyclophellitol β-epoxide MDW933

**Fig 1:**
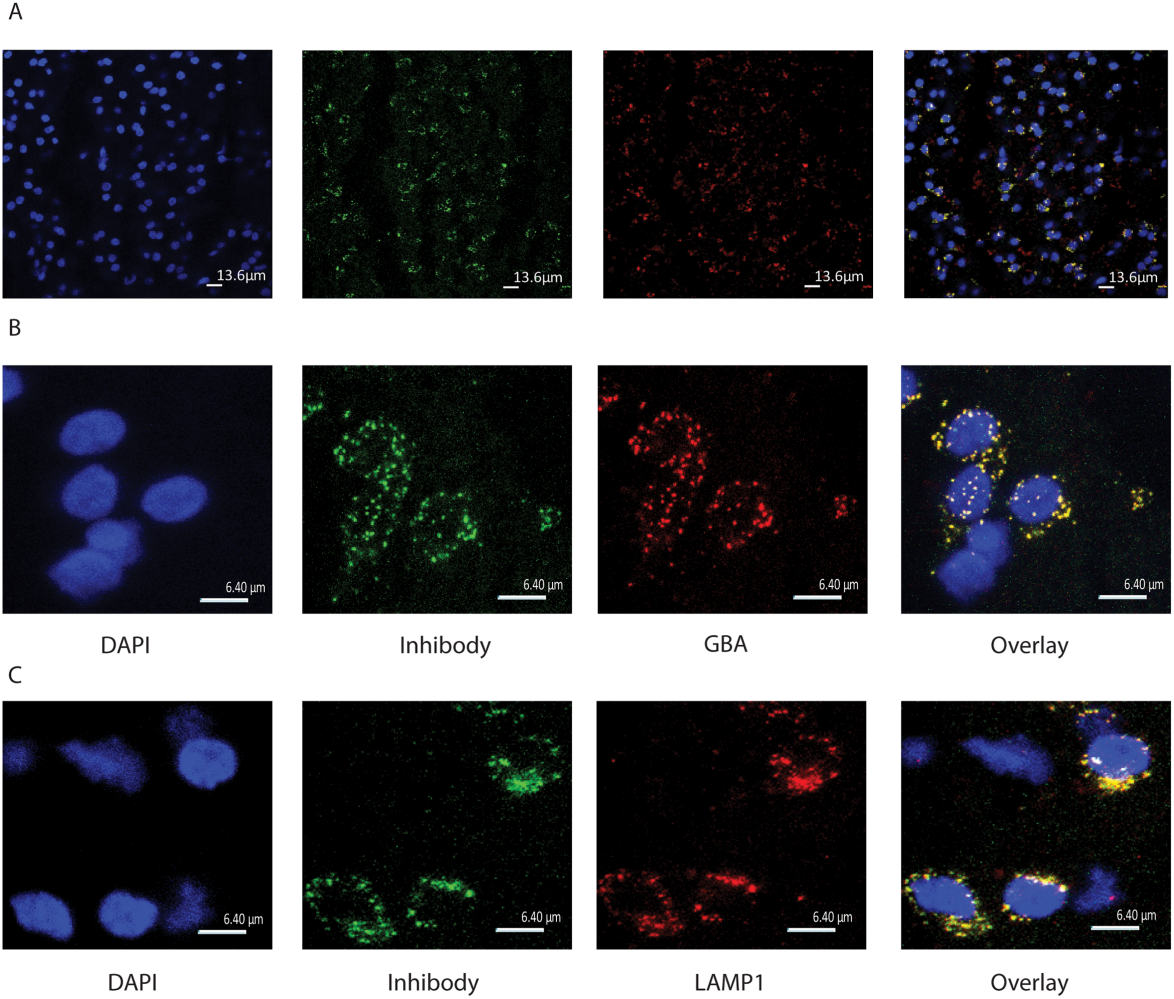
Cyclophellitol-epoxide ABP MDW933 administered i.c.v. labels specifically lysosomal GBA in the brain. (A) 63x Confocal images showing from left to right: DAPI stain, MDW933 (green Inhibody) labeling, GBA antibody labeling (red) and their overlay in the Po of the brainstem. (B) Zoom (3x) 63x confocal images showing clear colocalization. (C) Zoom (3x) 63x confocal images showing from left to right: DAPI stain, green MDW933, the lysosomal protein LAMP1 antibody labeling (red) and their overlay in the Po of the brainstem.

To fluorescently label active GBA molecules in the brain, *in vivo* i.c.v. infusions with cyclophellitol β-epoxide ABPs were performed in living rats. Firstly, we infused Wistar rats i.c.v. with green fluorescent MDW933 (Inhibody green) for 10 minutes. Four hours after the infusion, animals were sacrificed, their brain was collected, sliced, and the presence of ABP signal was studied by fluorescence microscopy (Fig 1). GBA antigen in slices was also visualized by immunohistochemistry with anti-GBA antiserum. The fluorescent ABP showed a lysosomal signal that almost completely overlapped with GBA antigen staining (Fig 1a and 3x zoom 1b). Immunohistochemical staining of lysosomal LAMP1 (Fig 1c) revealed that the ABP signal is largely localized in lysosomes.

**Fig 2:**
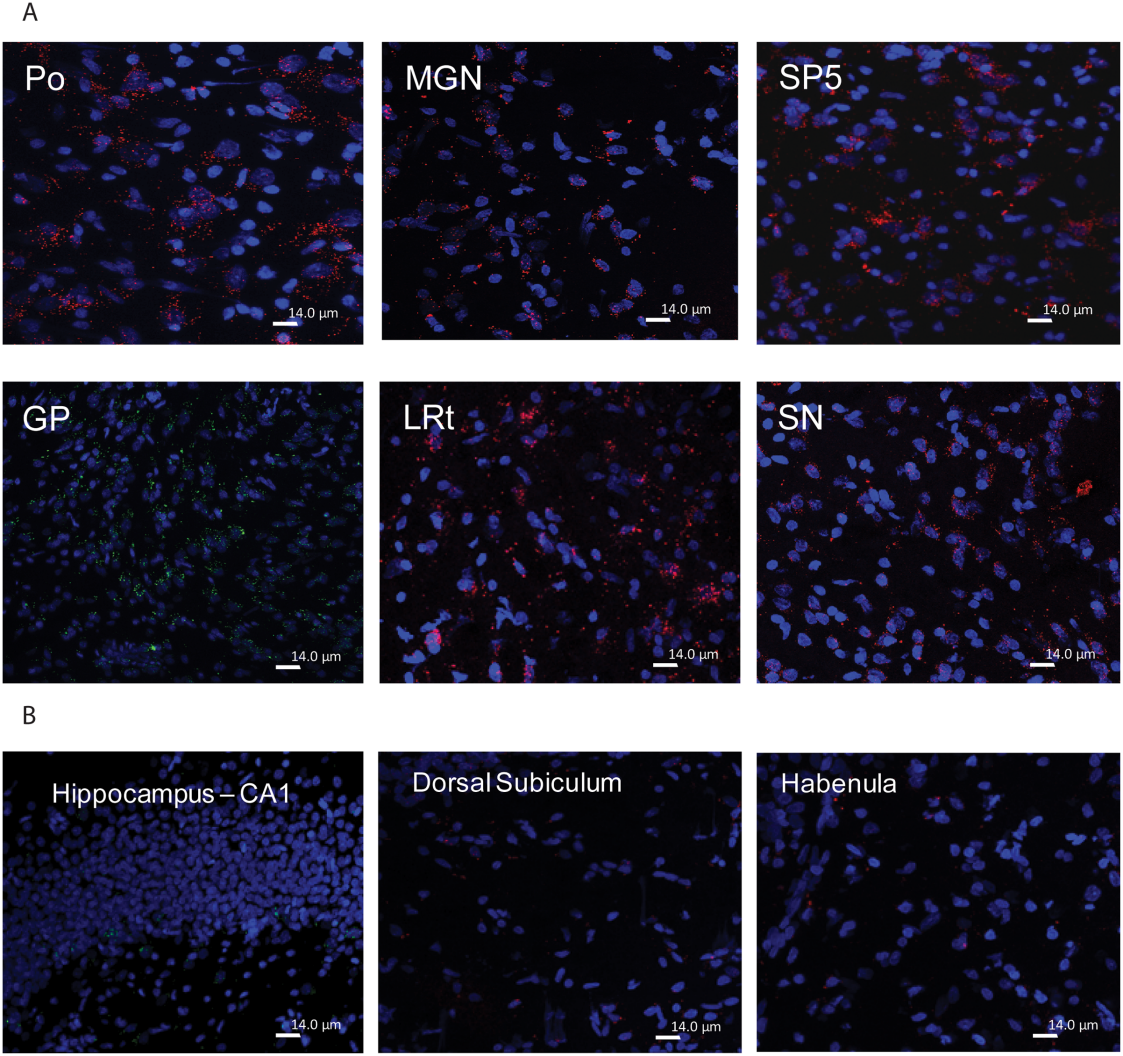
Prominent GBA-labeling by infused inhibody ABPs in motor related areas of the brain. (A) Strong red MDW941 labeling of brainstem motor related structures Po, SP5, Lrt, SN and MGN after i.c.v. administration. Example of green MDW933 labeling of GP (middle panel left). (B) Modest labeling of motivational and memory processing areas. Example of modest MDW933 labeling of hippocampus (left panel) and modest MDW941 labeling of dorsal subiculum, and habenular (middle and right panel).

We next identified areas of rat brain with high and low concentrations of GBA molecules as labeled with cyclophellitol-epoxide type ABPs MDW933 (Inhibody green) or MDW941 (Inhibody red). With both ABPs similar results were obtained. Fig 2a shows that motor related areas such as basal ganglia (GP and SN), the medial geniculate nucleus (MGN) and brainstem motor related structures such as the spinal trigeminal nucleus (SP5), lateral reticular nucleus (Lrt) and Po contain relative high concentrations of active GBA molecules compared to other structures. Motivational and memory related brain areas appear to contain relatively lower concentrations of active GBA molecules, particularly the hippocampal CA1, CA2, and CA3, the dorsal subiculum and the habenular nucleus (Fig 2b). It should be mentioned that the hippocampus is known to express high P-glycoprotein activity (see for an example [31]). BODIPY-containing compounds, like the ABPs used, are known to act as substrates for P-gycoprotein mediated efflux from the brain. Therefore it is possible that the relatively modest labeling of GBA in the hippocampus is due to low local ABP concentrations.

**Fig 3:**
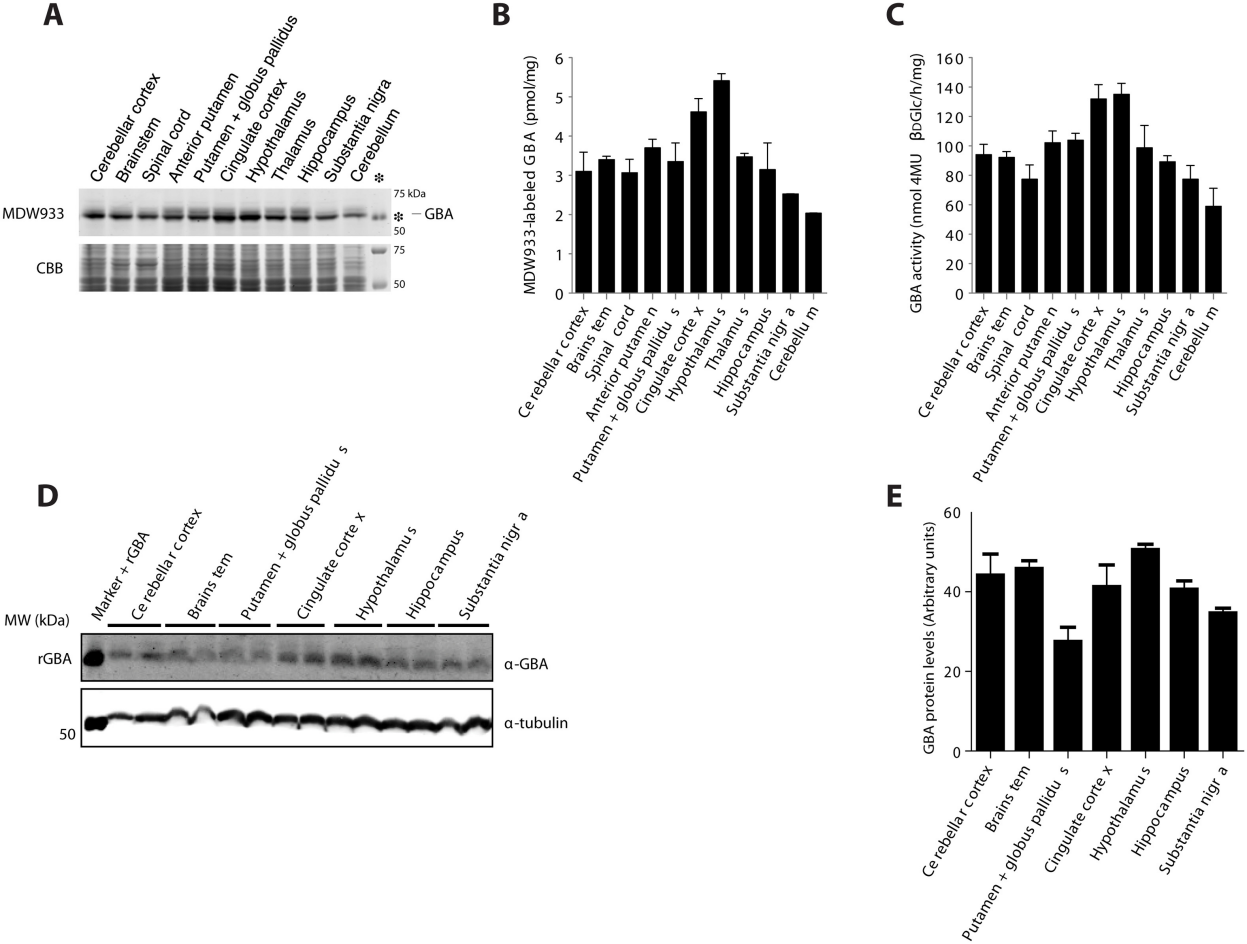
Active GBA in specific brain areas. (A) SDS-PAGE gel of Inhibody MDW933 labeled rat brains areas. The asterisk indicates positive control recombinant GBA signal. As a loading control the same gel was stained with Coomassie brilliant blue (CBB). (B) Quantification of fluorescence expressed per μg of total protein. (C) Enzymatic activity of GBA in brain areas, as determined with 4MU-β-d-glucopyranoside substrate. (D) GBA protein levels in homogenates of rat brain areas assessed by Western blot analysis. (E) Quantification of GBA blot in rat brain areas normalized to tubulin.

Next, punches of specific brain areas of two untreated rats were isolated and homogenates thereof were incubated with 100 nM green fluorescent Inhibody MDW933. Labeled GBA was visualized by SDS-PAGE gel electrophoresis and fluorescence scanning, while applying per lane identical amounts of total protein. As shown in Fig 3a and 3b, the hypothalamus and the cingulate cortex seem to contain relatively high amounts of active GBA molecules per total protein as determined with the BCA assay. Similar results were obtained with materials of two rats. The brainstem, thalamus, basal ganglia and hippocampus appear to contain slightly smaller amounts of active GBA per total protein. Next, we determined the enzymatic activity of GBA per total protein in the same punches (Fig 3c). A similar pattern for active GBA was noted by ABP labeling and measurement of enzyme activity. It should be pointed out that relating ABP labeling (or enzymatic activity) to total protein as determined by the Pierce BCA assay is to some extent misleading. Comparing ABP labeled GBA with Coomassie Brilliant Blue stained protein in the same fraction (see Fig 3a) suggests that the differences in GBA level between regions are relatively small.

Additional Western blot analysis of GBA antigen showed that the same brain areas contain similar GBA protein levels, except for the Globus pallidus + Putamen, which seem to show less GBA protein content (Fig 3d and 3e).

**Fig 4:**
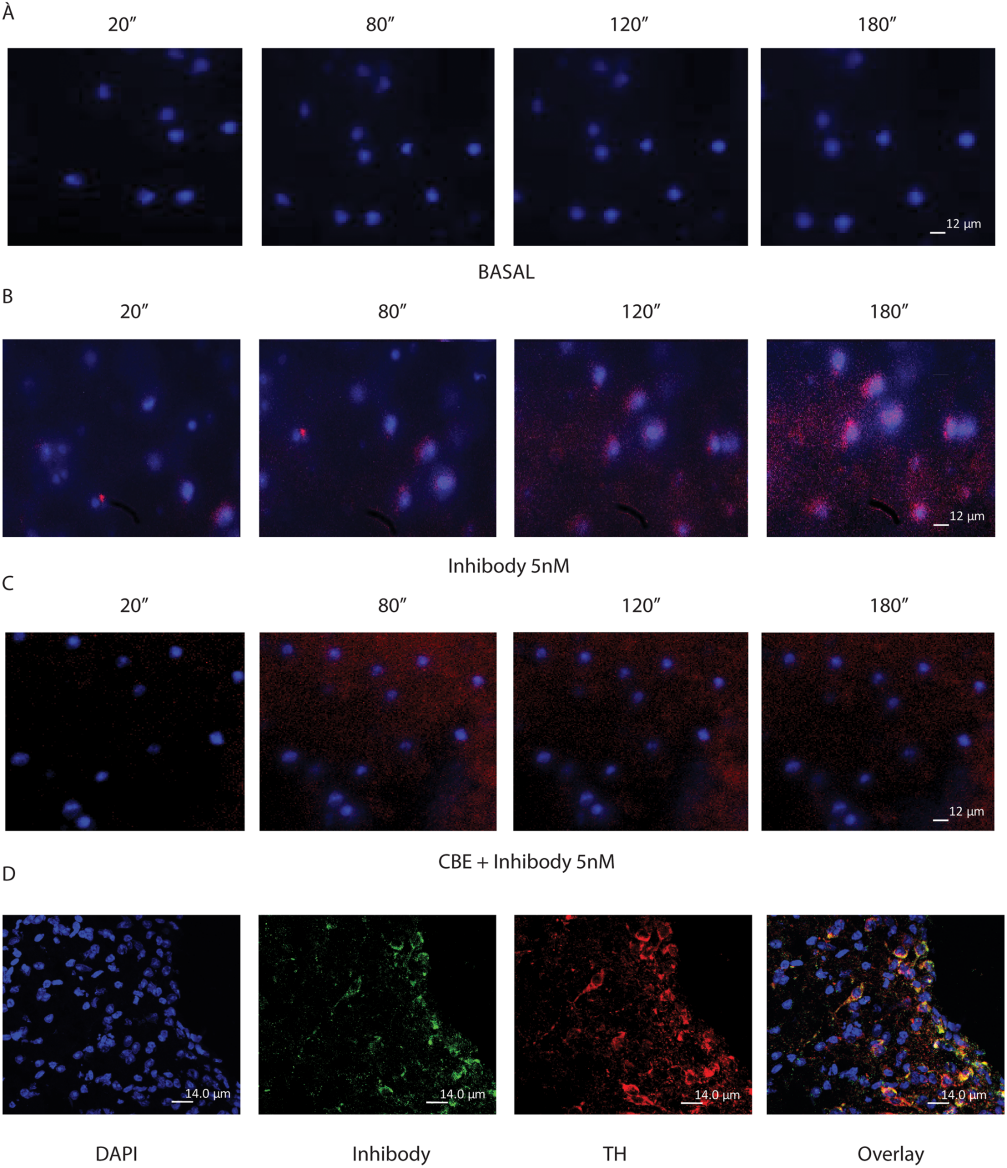
*Ex vivo* live imaging of GBA labeling in the SN. (A) Ex vivo live imaging of DAPI-stained SN during 10 minutes with a 20 second interval per picture. (B) Ex vivo live imaging of the SN after 5 nM MDW941 administration to the media. (C) Ex vivo live imaging of the SN after incubation with CBE followed by Inhibody addition to the media. (D) TH antibody staining of SN slices after ABP labeling.

To confirm specific GBA labeling in brain, we also performed *ex vivo* time-lapse analysis of the SN of mice (Fig 4). Time-lapse imaging of slices incubated with red fluorescent Inhibody MDW941 (5 nM) alone showed perinuclear labeling typical for lysosomes (Fig 4b). Slices incubated with cerebral spine fluid (CSF) as control lacked this signal (Fig 4a). Pre-incubation of slices with CBE, a known irreversible inhibitor of GBA, blocked fluorescent labeling with MDW941 [15], thus prevented labeling of any lysosomal structures (Fig 4c). Tyrosine hydroxylase positive dopaminergic neurons showed high amounts of active GBA labeling (Fig 4d). Dopaminergic neurons in the SN are involved in the output of motor movement and coordination and are affected in α-synucleinopathies like Parkinson disease (PD) [19].

**Fig 5:**
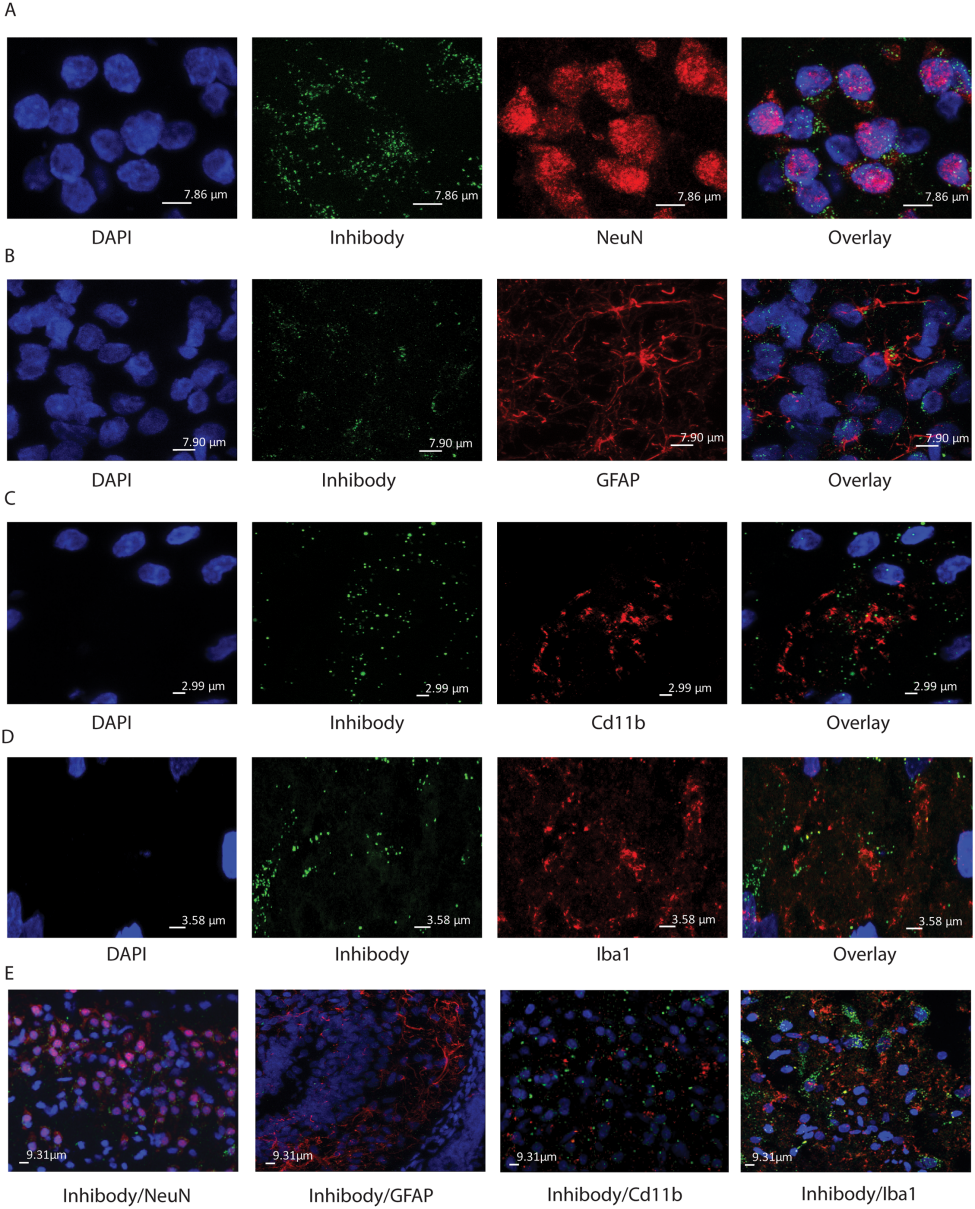
Presence of active GBA in neurons. Shown is zooming (3×) of 63× confocal images in A-C. (A) SN of i.c.v. infused rats with MDW933. From left to right images of DAPI, green MDW933, red NeuN antibody staining and overlay image. The overlay shows the Inhibody label around NeuN positive nuclei of neuronal cell suggesting lysosomal localization. (B) Median Eminence (ME) of i.c.v. infused rats with MDW933. From left to right images of DAPI, green MDW933, red GFAP antibody and overlay image. The overlay shows GFAP positive cells with concomitant Inhibody labeling. (C) SN of i.c.v. infused rats with MDW933. From left to right images of DAPI, green MDW933, red CD11b antibody and overlay image. The overlay shows MDW933 signal in areas containing microglia cells. (D) SN of i.c.v. infused rats with MDW933. From left to right images of DAPI, green MDW933, red Iba1 antibody and overlay image. The overlay shows MDW933 signal in areas containing microglia cells. (E) 63x confocal images of the SN and ME of i.c.v. infused rats with MDW933. The images show from left to right green MDW933 and red NeuN antibody (SN); green Inhibody and red GFAP antibody (ME); green Inhibody and red CD11b antibody (SN); green Inhibody and red Iba1 antibody (SN).

To investigate more closely which cell types of the brain present low and high amounts of active GBA molecules, we stained brain sections from Inhibody MDW933-infused rats for different markers of microglia (Cd11b and Iba1), astrocytes (GFAP) and neurons (NeuN). Histological analysis of basal ganglia and brainstem motor related structures revealed that active GBA is abundantly present in neurons marked by presence of NeuN (Fig 5a and 5e). Active GBA is also present in astrocytes and microglia. In the astrocyte-rich median eminence (ME), GFAP positive cells were labeled by ABP MDW933, although less prominently than NeuN positive cells (Fig 5b and 5e). The same was observed for the microglia (CD11b positive and Iba1 positive) in different motor areas like the SN (Fig 5c, 5d and 5e).

**Fig 6:**
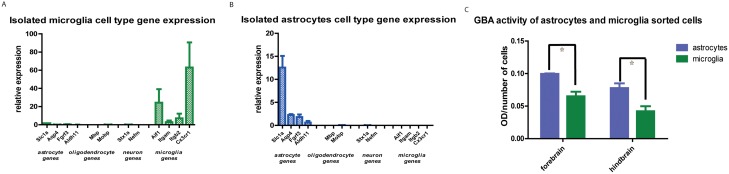
GBA in sorted CD11b positive microglia and GLT1 positive astrocyte populations. (A) RT PCR mRNA expression of CD11b positive sorted cells. (B) RT PCR mRNA expression of GLT1 positive sorted cells. (C). Quantification of green MDW933-labeled GBA in microglia and astrocytes expressed per number of cells.

Next, microglia and astrocytes were isolated from forebrain (hippocampus, anterior basal ganglia and cortex), and hindbrain (combined midbrain and brainstem areas) from rats. Astrocytes were purified by FACS sorting based on GLT1, a glutamate transporter expressed specifically in astrocytes widely throughout the brain. Microglia cells were sorted based on CD11b expression. Due to the high autofluorescence of microglia, a special gating of the 7AAD positive signal for the microglia populations was used [30]. The FACS procedure gave clear separation of the GLT1 positive/CD11b negative population (astrocytes) and the CD11b positive population (microglia). We performed RT PCR for astrocyte markers in the GLT1 and CD11b positive populations. The GLT1 positive cell population showed a higher mRNA expression of genes prominently expressed in astrocytes: Slc1a2, Aldh1l1, Aqp4 and Fgfr3, and low or no mRNA expression of genes prominently expressed in microglia: Aif1, Itgam, Itgb2 and Cx3cr1 (Fig 6a and 6b). CD11b positive cell populations showed a high mRNA expression of genes mostly expressed in microglia: Aif1, Itgam, Itgam and Cxc3r1, especially of Cxc3r1 being the highest expressed in CD11b positive cell populations (Fig 6a and 6b). To additionally confirm the purity of the astrocyte and microglia populations, we analyzed the mRNA expression of genes that are mostly expressed in neuronal cells and oligodendrocytes. Both GLT1 and CD11b positive populations showed no mRNA expression of genes expressed in neurons (Stx1a and Nefm) and no mRNA expression of genes expressed in oligodendrocytes (Mbp and Mobp) (Fig 6a and 6b). GBA in sorted astrocyte and microglia cell population was labeled with 200 nM MDW933. Astrocytes were found to contain more active GBA per cell compared to microglia cells (p<0.003) (Fig 6c).

### Labeling of active GBA2 with aziridine-cyclophellitol ABP

**Fig 7:**
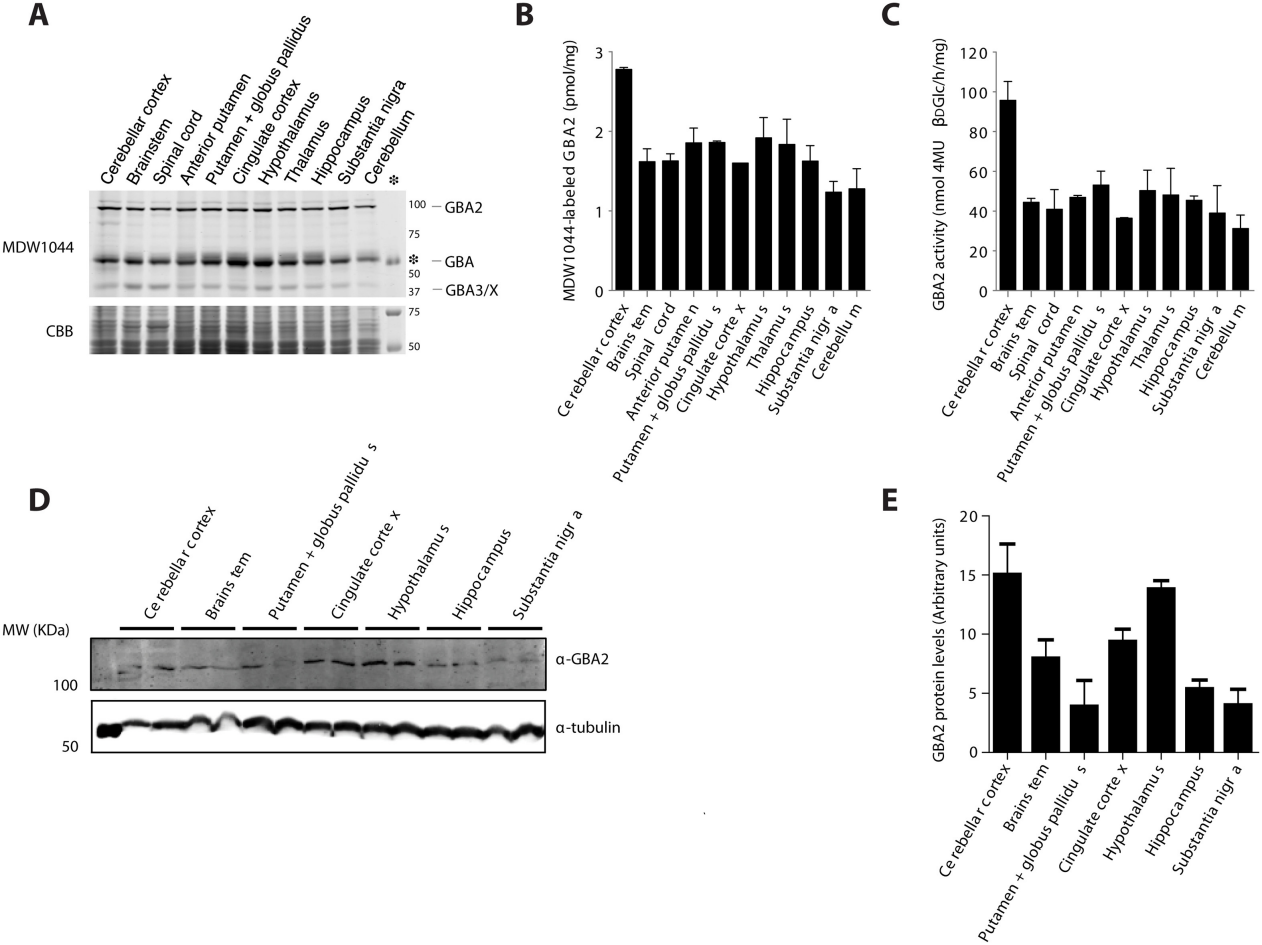
*In vitro* labeling of GBA2 in specific brain areas. (A) SDS-PAGE gel of Anybody MDW1044 labeled rat brains areas. (B) Quantification of Anybody MDW1044 fluorescent signal per μg of protein. (C) GBA2 enzymatic activity in different brain areas. (D) GBA2 protein levels in homogenates of rat brain areas assessed by Western blot analysis. (E) Quantification of GBA2 blot in rat brain areas normalized to the same tubulin signal showed in Fig 3e. Note: cerebellum indicates other parts of cerebellum than cerebellar cortex.

We next employed the β-aziridine cyclophellitol ABP which broadly target β-glucosidases [23]. It was first examined whether brain GBA2 can be labeled with these Anybody ABPs. For this purpose, we incubated punches of specific brain areas of untreated rats *in vitro* with 100 nM green fluorescent Anybody MDW1044 (Fig 7). Following gel electrophoresis and fluorescence scanning, three distinct proteins become visible: GBA2 with MW ~100 kDa, GBA with MW ~60 kDa and a protein of about 40 kDa.

The enzyme GBA2 was detected in all regions (Fig 7a and 7b). A similar result was obtained by measuring GBA2 activity in homogenates of the punches (Fig 7c). The cerebellar cortex showed the highest amount of active GBA2 with both methods. Similar findings were made by Western blot analysis of GBA2 antigen, in which the cerebellar cortex and hypothalamus showed higher levels of GBA2 (Fig 7d and 7e).

**Fig 8:**
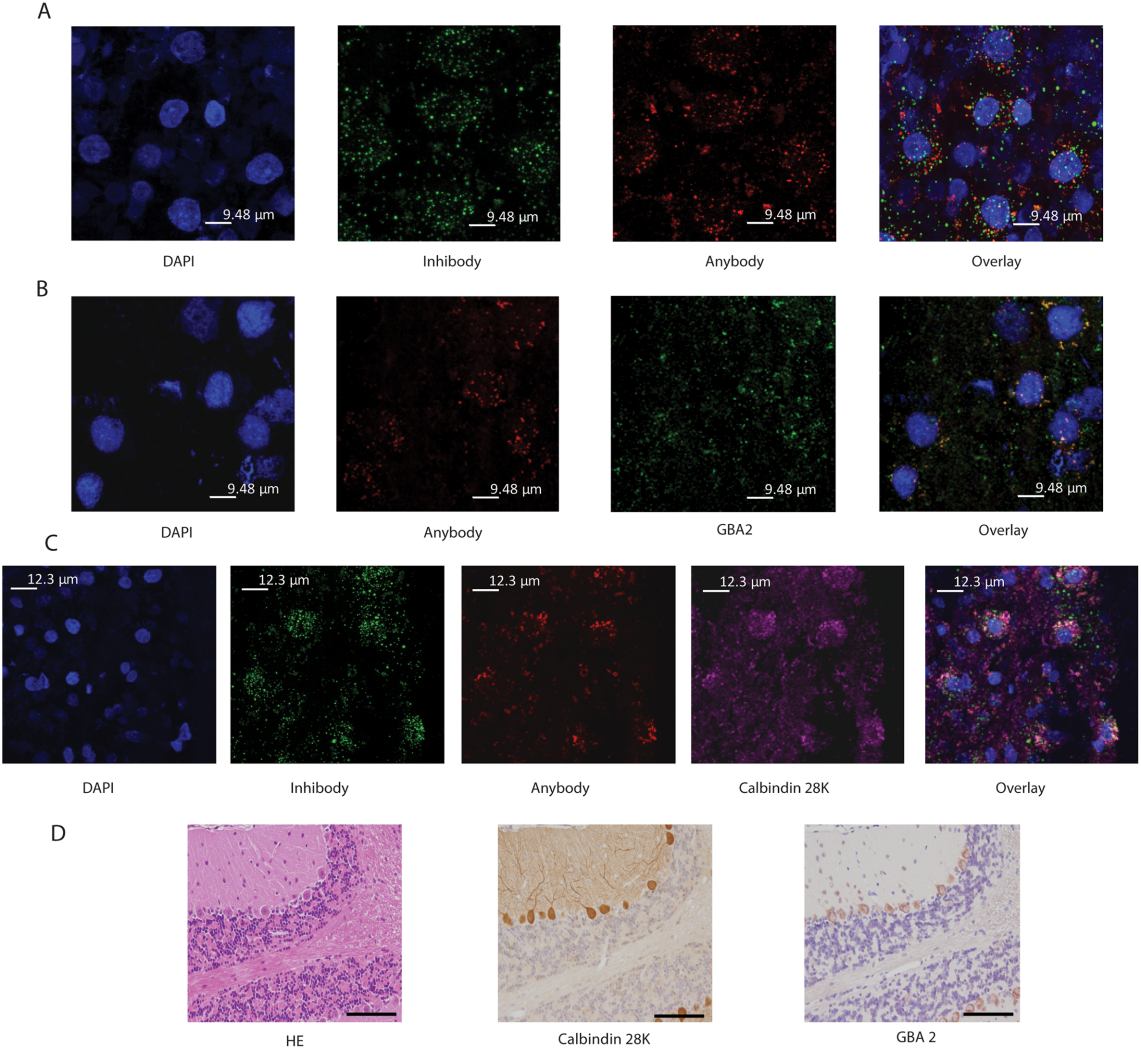
Labeling of cerebellar cortex with β-aziridine cyclophellitol ABP JJB75. (A) Zoom (3x) of 63x confocal images of the cerebellar cortex of green Inhibody MDW933 > red Anybody JJB75 i.c.v. infused animals. From left to right images of DAPI, green MDW933 signal, and red JJB75 signal are shown. The overlay shows that Inhibody and Anybody signals colocalize to only a minor extent. (B) Zoom (3x) of 63x confocal image. Cerebellar Cortex of CBE > red Anybody JJB75 i.c.v. infused animals. From left to right images of DAPI, red JJB75, anti-GBA2 antibody signal (green) and overlay image are shown. (C) Zoom (3x) of 63x confocal images of the cerebellar cortex of Inhibody MDW933 > Anybody JJB75 i.c.v. infused animals. From left to right images of DAPI, green MDW933, red JJB75, Calbindin 28K antibody staining (magenta) and overlay image are shown. (D) GBA2 presence in Purkinje cells in mouse brain. From left to right: HE staining, Calbindin 28K antibody staining and anti-GBA2 antibody staining in separate sections.

To study more closely other enzymes in the cerebellar cortex than GBA being labeled by Anybody ABP, we first blocked GBA for Anybody labeling by prior i.c.v. infusion with Inhibody or CBE. Rat brains were i.c.v. infused with green fluorescent Inhibody MDW933 followed by red Anybody JJB75 or the combination CBE followed by JJB75. Microscopic analysis of the cerebellar cortex from rats sequentially infused with green Inhibody MDW933 followed by red Anybody JJB75 showed both green and red fluorescence within the same neuronal cells (Fig 8a). The distribution of red Anybody labeling was similar in brains of animals firstly infused with CBE (Fig 8b). We noted prominent red Anybody labeling in the cerebellar cortex of Calbindin 28K positive Purkinje cells (Fig 8c). The nature of enzymes labeled with Anybody additionally to GBA is not yet fully clear. Aside from GBA, GBA2 and GBA3 also label with β-aziridine cyclophellitol ABPs [17], and currently labeling of other enzymes cannot be completely excluded. Of note, prominent presence of GBA2 in Purkinje cells of mice has indeed been demonstrated by antibody analysis (Fig 8d).

## Discussion

So far, no methods exist for visualizing active GBA molecules in the intact brain. We earlier developed β-glucopyranosyl-configured cyclophellitol-epoxide ABPs specifically labeling active GBA in intact cells and viscera of mice [21]. In this study we firstly demonstrate that i.c.v. administration of such ABPs can be used to detect with high spatial resolution active GBA molecules in the brain. Our findings regarding the distribution of active GBA molecules in the brain deserve discussion in view of the documented association of impaired GBA and α-synucleinopathies [5–14]. We observed with our ABP-based labeling method that the basal ganglia structures like the SN, GP and MGN, and brainstem structures like Po, SP5 and Lrt present high levels of active GBA. These particular structures are involved in motor function and form part of the extrapyramidal system of motor coordination. Our finding of relative high amounts of active GBA in these motor related areas in the healthy brain may point to an important local role for the enzyme, consistent with the susceptibility of such areas to develop Lewy Bodies during partial GBA deficiency in GD carriers and patients [8]. Various hypotheses have been put forward to link glucocerebrosidase abnormalities to α-synuclein accumulation. For example, it has been proposed that the enzyme directly interacts with α-synuclein [32, 33] and mutant enzyme has been found to be present in Lewy bodies [34]. In addition, it has been proposed that increased α-synuclein disrupts intracellular trafficking of GBA resulting in a positive feedback loop amplifying the pathologic effects of α-synuclein [12]. A different hypothesis proposes disturbance of proteasome function following ER-associated degradation of mutant enzyme [35].

Of interest, other brain areas found to be rich in active GBA are the hypothalamus and cingulate cortex. This finding was confirmed by analysis of GBA in dissected areas of brain. Recently metabolism of ceramide and glucosylceramide in the hypothalamus has been proposed to play an important role in control of energy homeostasis [36–38]. The prominent ABP-labeling of active GBA in the hypothalamus is in line with these observations and future studies with i.c.v. administrations of fluorescent ABPs may shed light on the role of hypothalamic GBA in the regulation of energy homeostasis.

Conceivable is future use of the here-described procedure for in vivo labeling of GBA in brain to investigate the effects of pharmacological agents. The efficacy of agents aiming to increase GBA levels in the brain can be studied using saturating ABP labeling. In theory, one could attempt to analyze the interaction of pharmacological agents with the GBA catalytic pocket by demonstrating competitive inhibition of ABP labeling. For this purpose, conditions would first need to be carefully established at which ABP labeling is not yet saturating.

A discussion of our findings in view of neuropathic GD is warranted. In the neuropathic variants of Gaucher disease neuronal accumulation of glucosylceramide occurs. The relation between lipid accumulation and neuropathology is still not completely understood. Recent studies with mouse models have led to interesting novel insights. Firstly, studies by Futerman and colleagues revealed that neuronal loss induced by exposure of mice to CBE, a brain-permeable suicide inhibitor of GBA, stems not from apoptosis but rather necrosis mediated by RIP3K [39]. Recently, the same research group also studied GBA-deficient neuropathic Gaucher disease (nGD) mice on the relationship between neuropathology and accumulation of glucosylceramide and glucosylsphingosine, the deacylated form of the lipid [40]. Four distinct areas were dissected from brains: the cortex and the ventral posteromedial/posterolateral (VPM/VPL) region of the thalamus (areas displaying significant pathology), and the caudate putamen (CPu) and the periaqueductal gray (PAG) (areas not displaying pathology). Interestingly, no strict correlation between GlcCer accumulation and residual GBA activity, as measured *in vitro*, was noted. GlcCer was found to accumulate comparably in the various brain regions, preceding accumulation of glucosylsphingosine. Only glucosylceramide accumulation was found to correlate with neuroinflammation and neuron loss. It was concluded that in the nGD mouse model a certain level of neuronal glucosylceramide storage is required to trigger neuropathological changes in affected brain areas, while other brain areas containing similar glucosylceramide levels are unaltered. Our analysis of the distribution of active GBA molecules across the healthy rodent brain does also not point to a simple match of GBA abundance in brain with the noted neuropathology in nGD mice. Areas like the cortex and thalamus displaying prominent pathology in nGD mice do not show an exceptional content on active GBA molecules in normal rat brain. It is attractive to conclude, similar to Futerman and colleagues [40] that beyond the primary GBA defect intrinsic differences in neuronal properties or in the neuronal environment between various brain regions determine onset of GD neuropathology. One such modulating factor could be the second glucocerebrosidase GBA2 [41,42]. Prominent ABP labeling of GBA2, confirmed by antibody staining, is seen for the cerebellar cortex, specifically in Purkinje neurons. Recent reports have indicated a clear association between GBA2 mutations and the development of cerebellar disorders and motor neuron neurodegeneration [43,44]. Our investigation revealed that in normal rat brain Purkinje cells in the cerebellar cortex present the highest GBA2. Of interest, also the thalamus appears rich in GBA2. In fact, the brain distribution of GBA2, being prominent in cortex and also rich in the thalamus, mimics more closely the areas involved in neuropathology in nGD mice as that of GBA.

Our novel method of ABP-based labeling of glucocerebrosidase in the brain offered also some insight in the types of cells expressing high levels of active GBA molecules *in vivo*. We observed that especially neurons present high amounts of active GBA. Labeling of active GBA in astrocytes and microglia is less pronounced. Astrocytes and microglia populations isolated from forebrain and hindbrain structures were examined *ex vivo* on GBA content. Astrocytes were found to have higher GBA activity than microglia, especially in the case of forebrain structures. Of interest, astrocytes are known to also develop Lewy Bodies [45]. Somewhat surprising is the observation of a relative low content of GBA in isolated microglia. Neuroinflammation and the presence of activated microglia in the areas with major neuronal cell death are thought to be major factors promoting neuronal cell death in alpha-synucleinopathies and neuronopathic GD mouse models [46,47].

In conclusion, when infused i.c.v., fluorescent β-glucopyranosyl cyclophellitol-epoxide ABPs can visualize active GBA molecules in the brain and thus help to increase insight in the role of this enzyme in neurodegenerative disorders. The β-glucopyranosyl cyclophellitol-aziridine type ABPs label next to GBA also other glycosidases like GBA2 and can be used to establish the distribution of active molecules of various enzymes along the brain. The relative high expression of GBA and GBA2 in neuronal cells in specific motor areas of the brain seems to correlate with motor related neurological disorders associated with their deficiencies such as Parkinson disease and cerebellar ataxia, respectively.

## Supporting Information

S1 File ARRIVE guidelines checklist. (PDF)
